# An Examination of COVID-19 Mitigation Efficiency among 23 Countries

**DOI:** 10.3390/healthcare9060755

**Published:** 2021-06-18

**Authors:** Emily Chia-Yu Su, Cheng-Hsing Hsiao, Yi-Tui Chen, Shih-Heng Yu

**Affiliations:** 1 Graduate Institute of Biomedical Informatics, College of Medical Science and Technology, Taipei Medical University, Taipei 11031, Taiwan; emilysu@tmu.edu.tw; 2 Clinical Big Data Research Center, Taipei Medical University Hospital, Taipei 11031, Taiwan; 3 Department of Health Care Management, College of Health Technology, National Taipei University of Nursing and Health Sciences, Taipei 11219, Taiwan; alvul4@hotmail.com; 4 Department of Business Management, National United University, Miaoli 36003, Taiwan

**Keywords:** COVID-19, stay-at-home order, mitigation efficiency, epidemic stability

## Abstract

The purpose of this paper was to compare the relative efficiency of COVID-19 transmission mitigation among 23 selected countries, including 19 countries in the G20, two heavily infected countries (Iran and Spain), and two highly populous countries (Pakistan and Nigeria). The mitigation efficiency for each country was evaluated at each stage by using data envelopment analysis (DEA) tools and changes in mitigation efficiency were analyzed across stages. Pearson correlation tests were conducted between each change to examine the impact of efficiency ranks in the previous stage on subsequent stages. An indicator was developed to judge epidemic stability and was applied to practical cases involving lifting travel restrictions and restarting the economy in some countries. The results showed that Korea and Australia performed with the highest efficiency in preventing the diffusion of COVID-19 for the whole period covering 105 days since the first confirmed case, while the USA ranked at the bottom. China, Japan, Korea, and Australia were judged to have recovered from the attack of COVID-19 due to higher epidemic stability.

## 1. Introduction

The COVID-19 pandemic has been raging across the world since the beginning of 2020, resulting in a substantial death toll. As of 30 May 2021, the World Health Organization (WHO) indicated that more than 216 countries, areas, and territories were found to have more than 169 million confirmed cases associated with 3.53 million deaths [1]. When the epidemic broke out, the governments of most countries in the world adopted various response strategies including viral tests to identify infected persons, wearing masks and practicing social distancing to prevent infection, and closing schools and businesses, etc., to slow the spread of the epidemic. After a period of hard work, the epidemic situation in some countries significantly improved, while in others, it was still deteriorating. The number of daily new confirmed cases in some countries has fallen to single or double digits, but in some other countries, it has not reached its peak and continues to increase. This shows that the response strategies adopted in each country may have different effects on the mitigation of COVID-19 transmission. 

How different response strategies affect the mitigation of new confirmed cases was analyzed by several studies. For example, Ding and Li [2] discussed the performances of various response strategies in fighting against COVID-19. Patrikar et al. [3] presented a mathematical model to predict the peak of the epidemic based on different levels of response strategies such as social distancing. Chen et al. [4] examined the effectiveness of transmission mitigation in association with the response strategies. However, very few studies focus on the analysis of the epidemic mitigation efficiency.

In this paper, we attempt to measure the relative efficiency in preventing the spread of COVID-19 using the data envelopment analysis (DEA) technique. In practice, the DEA technique has been widely used in various applications, including health industries [5,6], energy sectors [7,8,9], cement industries [10], agricultural production [11,12], and manufacturing sectors [13], and it has proven to be an effective approach in identifying the best practice frontiers.

In the field of medical services, DEA was also widely used to measure the efficiency of hospitals in association with patient visits, surgeries, and discharges. For example, Khushalani and Ozcan [14] employed a dynamic network DEA to examine the efficiency of production quality in hospitals and found that urban and teaching hospitals were less likely to improve quality production efficiency. Deily and McKay [15] used efficiency scores obtained from a DEA analysis as explanatory variables to determine hospital efficiency. In other fields, Oggioni et al. [10] employed DEA to analyze efficiency by using energy as an input and one desired output accompanied by undesired outputs (CO_2_ emissions). Mousavi-Avval et al. [11] and Mohammadi et al. [12] applied the DEA technique to measure the efficiency of agricultural production to identify wasteful energy. Vazhayil and Balasubramanian [9] showed that the weight-restricted stochastic DEA method was appropriate to optimize power sector strategies.

To compare the mitigation efficiency among countries on a fair basis, the time period for each stage was calculated from the date of the first confirmed case in each country. The whole period covers 105 days from the first confirmed case and was divided into six stages. In addition to the measurement of overall efficiency covering 105 days, the efficiency at each stage was also evaluated. Firstly, the purpose of this article was to compare the relative efficiency of each country in mitigating the spread of the COVID-19 epidemic. Secondly, the trends of efficiency rank across stages for each country were analyzed. Eventually, an indicator for epidemic stability was developed to judge the status of epidemic stability for each country.

## 2. Research Methods

To compare the relative efficiency in preventing and reducing the spread of COVID-19, a total of 23 countries were selected, including 19 countries in the G20 and four other representative countries, as listed in Table 1. The reason for the selection of Iran and Spain was due to their high levels of confirmed cases and deaths. Pakistan and Nigeria were chosen due to their large populations, which reached 220.9 million and 206.1 million, respectively, at the end of 2020 [16].

The WHO [1] divided the stages of transmission into (1) no cases reported or observed (Stage 0); (2) imported cases (Stage 1); (3) localized community transmission (Stage 2); and (4) large-scale community transmission (Stage 3). As the date of the first confirmed case varied across countries, the period of each stage was not based on the same date among these countries but was calculated instead from the date of the first confirmed case in each country. The date of the first confirmed case was identified based on the daily situation report released by the WHO [1] starting on 21 January 2020. Among the 23 counties selected, China, Japan, and Korea reported having confirmed cases of COVID-19 before 21 January 2020. The information released from the WHO [1] demonstrated that some cases of pneumonia of unknown etiology were detected in Wuhan City, Hubei Province, China, on 31 December 2019. On 7 January 2020, a new type of coronavirus was isolated and identified. Thus, the first case in China may be considered to have occurred at the end of 2019. According to the WHO [1], the first confirmed cases of COVID-19 in Japan and Korea were reported on 15 and 20 January 2020, respectively.

The overall efficiency was compared based on the whole period covering 105 days since the first confirmed case for each country. The development process of COVID-19 spread was separated into 6 stages. As the number of new confirmed cases reported in earlier days is much lower, Stage 1 covers the first 30 days after the first confirmed case in each country. Each stage from Stage 2 to Stage 6 covered 15 days. The starting and ending dates of each stage for each country are listed in Table 1.

### 2.1. The DEA Model

In this paper, the DEA model was employed to measure the mitigation efficiency regarding the spread of COVID-19 at each stage for each country. The DEA model, proposed by Charnes et al. [17] based on the frontier production function defined by Farrell [18], is a nonparametric technique for measuring the relative efficiency of each decision-making unit (DMU) [19]. The mitigation of COVID-19 transmission in each country was executed by a technology whereby *N* countries in terms of DMUs transform a non-negative vector of multiple inputs, denoted x= ($x_{1}$, …, $x_{m}$)∈ ${ℜ}_{+}^{m}$, into a non-negative vector of multiple outputs, denote y= ($y_{1}$, …, $y_{s}$)∈${ℜ}_{+}^{s}$. This paper employed the basic DEA model of Charnes, Coopers, and Rhodes (CCR) to calculate the efficiency of COVID-19 transmission mitigation. The CCR model, under the hypothesis of constant returns to scale, is expressed as follows:

$$Min\begin{array}{cc} & \theta \end{array}$$

(1) $$\begin{array}{ll} s.t. & \theta \begin{array}{c} x_{0} \end{array}-X\lambda \geq 0\\ & Y\lambda \geq y_{0}\\ & \lambda \geq 0 \end{array}$$

where $y_{0}$ is the output, $x_{0}$ is the input, X and Y are the datasets in the matrices, λ is a semipositive vector, and θ represents the technical efficiency.

After the efficiency at each stage was obtained, Pearson correlation tests were conducted between the different stages at a *p*-value < 0.01 to examine the variation in efficiency ranks across stages. The correlation tests were used to explain the impact of the efficiency ranks at previous stages on subsequent stages. 

In this paper, epidemic stability (ES) is defined as the recovery status from the epidemic, and the indicator ES is presented by measuring the average increase in the proportion of confirmed cases to population (PCCP) during the period of the last day of Stage 6 and a day designated to restart the economy, expressed as follows:

(2) $$\mathrm{ES}=\frac{S_{f}-S_{0}}{\Delta t}$$

where $S_{f}$ and $S_{0}$ denote the PCCP on the last day of Stage 6 and the designated day, respectively, and Δt represents the period between the two dates.

### 2.2. The Variables

Efficiency, described as the relative performance regarding the reduction in COVID-19 transmission, was measured in this paper using the DEA method and is stated in the form of an output/input ratio. The objective of the authority administration was to minimize the total confirmed cases that occurred in each stage with a given amount of resources used. Cooper et al. [19] suggested that the DEA technique can be easily applied to a multiple input–output framework to compare the relative efficiency among various DMUs. The information produced from the DEA is valuable for identifying specific efficient units for future learning [20].

Neiderud [21] suggested that the rise of megacities may yield potential risks for new epidemics and become a threat in the world. The high human population density and close human-to-human contact are major sources for the rapid spread of respiratory diseases or avian flu. The growth and density of the human population may work as an incubator for infectious diseases, and urbanization as a driver of disease may have a negative effect on public health [22,23]. Thus, variables including (1) newly confirmed cases *n*, (2) population density *d*, and (3) urbanization degree *u* for each country were employed to measure the relative efficiency. As more confirmed cases represent less efficiency, newly confirmed cases *n* was treated as an input variable in Equation (1) to measure mitigation efficiency. In essence, the higher the population density and urbanization of a country are, the greater the chance of infection is. Thus, population density *d* and urbanization degree *u* were treated as output variables in Equation (1) for the measurement of mitigation efficiency.

### 2.3. Data Collection

The data for accumulated confirmed cases were extracted from the daily situation reports from the WHO [1], and the total confirmed cases in each stage were calculated by the difference in the accumulated confirmed cases on the last day of each stage and the previous stage. The population density data for each country were provided by Worldometer [24], and the urbanization degree data were extracted from the World Bank [16]. The descriptive statistics for the total accumulated confirmed cases across the 6 stages (i.e., 105 days since the first confirmed case), population density and urbanization degree are presented in Table 2. By the end of Stage 6 (i.e., 105 days since the first confirmed case), the USA had 1,193,452 confirmed cases, ranking at the top of the 23 countries, while Australia had the lowest number (6914) of confirmed cases. Korea had the highest population density at 527.30 persons per km^2^, while Australia had a much lower population density at 3.32 persons per km^2^. Argentina had the largest urbanization degree at 92% and ranked at the top. In contrast, the urbanization degree of India was much lower than the average of 71.48% based on the other countries and was only 34%.

The efficiency score was calculated through the assistance of the software DEA solver 13.

## 3. Results

**Figure 1:**
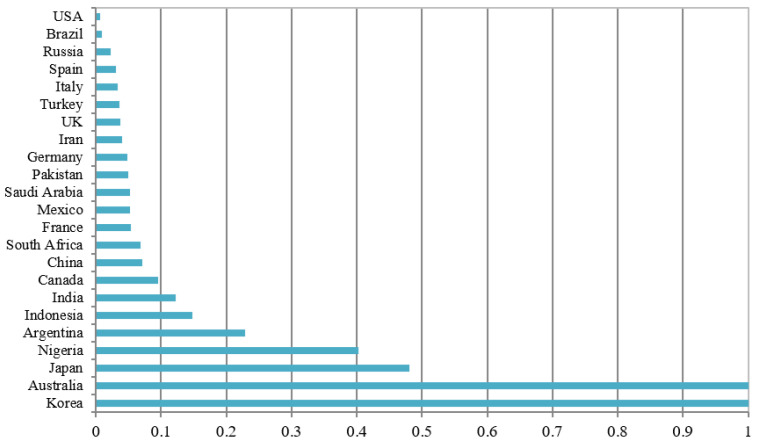
Mitigation efficiency scores among the 23 countries.

The efficiency of COVID-19 mitigation covering the first 105 days after a confirmed case for each of the countries is depicted in Figure 1. Australia and Korea rank at the top in terms of mitigation efficiency. In contrast, the USA ranks at the bottom, followed by Brazil and Russia. The major cause affecting the efficiency rank may be attributed to the number of total confirmed cases occurring over the whole period. The total confirmed cases in Australia and Korea in the whole period (covering 105 days since the first confirmed case) were only 6667 cases and 10,801 cases, respectively, while the USA, Brazil, and Russia had 1,193,452; 739,503, and 272,043 cases, respectively.

**Figure 2:**
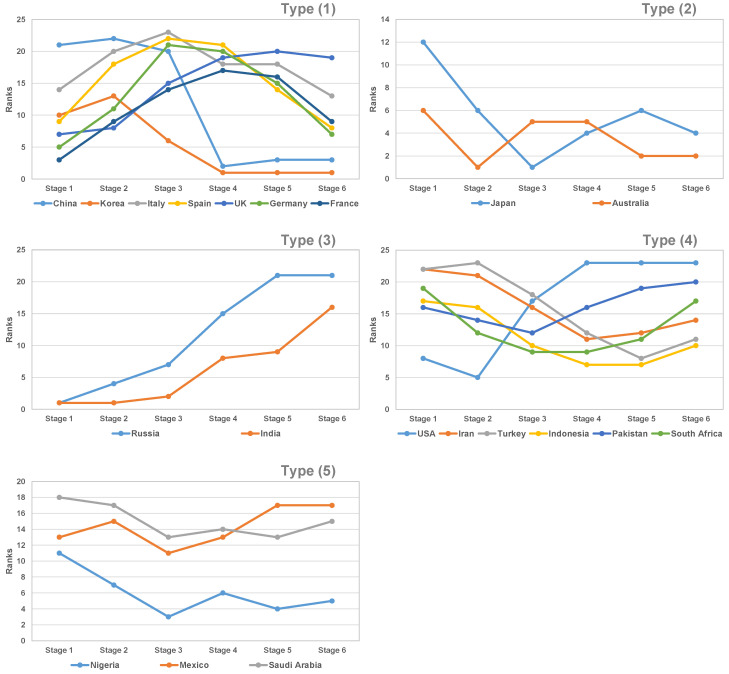
The trends in efficiency rank for countries of Types (**1**)–(**5**).

The efficiency scores and ranks at each stage for each country were also calculated according to Equation (1). Based on the shape of the efficiency ranking trend, these countries were classified into five types, as depicted in Figure 2.

### 3.1. Type (1): An Inverted U-Shaped Pattern Including Korea, China, Italy, Spain, UK, Germany, and France

This pattern in the efficiency rank trends was characterized by a continual decline in mitigation efficiency from Stage 1, which, after reaching the lowest point in the efficiency ranks, continued to improve until the last stage (Stage 6). Efforts to mitigate newly confirmed cases through the implementation of response strategies may have eventually achieved a certain effect. In essence, the mitigation efficiency in Type (1) gradually deteriorated in the middle stages. Passing through the peak of daily new confirmed cases, the COVID-19 transmission was then reduced, and the efficiency started to improve through the last stage. For example, Italy ranked 14th in Stage 1 and then dropped to 20th in Stage 2. Italy then reached a peak of daily confirmed cases, amounting to 6557 cases on 22 March 2020, which occurred in Stage 3. After Stage 3, the COVID-19 transmission in Italy improved, and the efficiency rank rose to 13th place in Stage 6. The efficiency ranks for China after Stage 3 and for Korea after Stage 2 showed great improvement and attained a relatively more stable state. China ranked 21st place and 22nd place at Stage 1 and Stage 2, respectively, but the efficiency rank was improved to 2nd place at Stage 4 and 3rd place at Stages 5 and 6 through a great number of emergency response strategies. Similar to China, Korea ranked in 10th place and 13th place for mitigation efficiency at Stages 1 and 2, respectively, and the efficiency improved to 6th place at Stage 3 and first place at Stage 4, which was subsequently maintained until the final stage. The other countries showed similar processes, but the degree of efficiency improvement was different.

### 3.2. Type (2): An Inverted N-Shaped Pattern Including Japan and Australia

In this type, the efficiency rank fluctuated across stages, with initial improvements followed by deterioration in the middle stages, but eventually, the efficiency rank improved in the final stages. For example, the efficiency rank for Japan improved continuously from 12th place in Stage 1 to 6th place in Stage 2 to first place in Stage 3, then dropped to 4th place in Stage 4 and 6th place in Stage 5, and eventually improved to 4th place again.

### 3.3. Type (3): Continual Decreases in Efficiency Rank Including Russia and India

The trend pattern in efficiency rank for Type (3) countries is characterized by the gradual deterioration in mitigation efficiency. The efficiency ranks are not bad in the earlier stages, but they worsen progressively. For example, Russia performed at the highest level regarding mitigation efficiency in Stage 1 and was ranked in first place. Unfortunately, Russia did not maintain this advantage, and its rank continued to deteriorate to 4th place in Stage 2 and, finally, to 21st place in Stage 6.

### 3.4. Type (4): U-Shaped Pattern Including the USA, Iran, Turkey, Indonesia, Pakistan, South Africa, Argentina, and Brazil

This trend in the efficiency ranks is characterized by some improvements in mitigation efficiency in the middle stages that eventually rebound back to a worse state. For example, the response in the USA to avoid COVID-19 transmission was not bad in Stages 1 and 2, as it ranked in 8th place and 5th place, respectively. However, its efficiency continually and dramatically dropped after Stage 2 and fell to 23rd place (the bottom of the ranking) in Stages 5 and 6. The efficiency improvement from Stage 1 to Stage 2 in the USA may be attributed to its prompt travel restrictions on China from 2 February 2020 and additional travel restrictions on Iran, Italy, and Korea on 29 February [25]. The gradual deterioration in efficiency ranking in the later stages in the USA implies that its response strategies may be ineffective for avoiding the epidemic.

The trend pattern in the efficiency ranking for Brazil provides a different story. From Stage 1 to Stage 6, the efficiency ranks for Brazil were not good. On 25 June 2020 (the final observation point in Stage 6) in Brazil, newly confirmed cases remained at a high level, amounting to 39,436 cases. This implies that the response strategies adopted by Brazil contained flaws.

### 3.5. Type (5): N-Shaped and W-Shaped Patterns Including Mexico, Nigeria, and Saudi Arabia

An N-shaped pattern for Mexico and W-shaped patterns for Nigeria and Saudi Arabia were identified. At the middle stages, the efficiency ranks for these Type (5) countries fluctuated very much. For example, Mexico ranked 13th place at Stage 1 and then dropped and rose in the middle stages, eventually dropping again to 17th place at Stage 6. As the efficiency for these two patterns drops again in the last stages, this implies that the mitigation efficiency is not stable and that the future trends for these countries are not optimistic.

To examine the impact of the efficiency rank at the previous stage on the subsequent stage, a Pearson correlation test of efficiency scores between different stages was conducted. The results are listed in Table 3. The correlation coefficient between Stage 1 and Stages 4–6 was very low, ranging from 0 to −0.1433. In contrast, the correlation coefficient was 0.788 between Stage 4 and Stage 5, 0.760 between Stage 4 and Stage 6, and 0.983 between Stage 5 and Stage 6. Table 3 also shows that the greater the distance is between any two stages, the lower the correlation coefficient is.

A numerical example is presented in this paper, in which it was proposed that the travel restrictions were lifted on the designed date of 27 June 2020; *ES*, $S_{f}$, $S_{0}$, and Δt were calculated according to Equation (2) for these 23 countries, and the results are listed in Table 4, where $S_{f}$ and $S_{0}$ are measured by cases per 100,000 persons, Δt in days, and *ES* by cases per 1,000,000 persons. The ranking of each country listed in Table 4 is based on the value of epidemic stability (ES).

Table 4 indicates that India has the lowest value of $S_{0}$ (PCCP in 105 days), amounting to 5.65 cases per 100,000 persons, a slightly lower value than that of China (5.81 cases per 100,000 persons). In contrast, Spain and Saudi Arabia have the highest values of $S_{0}$, amounting to 492.32 and 379.30 cases per 100,000 persons, respectively, which are much higher than the average of 172.19 cases per 100,000 persons. However, the ranking of the PCCP on 27 June 2020 ($S_{f}$) changes very much. China ranks at the top with the lowest $S_{f}$, amounting to 5.92 cases per 100,000 persons. The PCCP in India increases very much from 5.65 at $S_{0}$ to 36.88 cases per million at $S_{f}$. The USA has the highest value at $S_{f}$, amounting to 727.37 cases per 100,000 persons.

Table 4 also demonstrates that the *ES* in China, Japan, Korea, and Australia is much better than that in the other countries, amounting to 0.01, 0.46, 0.68, and 0.89 cases per million persons per day, respectively, during the period between the last day of Stage 6 and 27 June 2020. In contrast, the *ES* in Brazil, Saudi Arabia, South Africa, and the USA reaches 143.67, 93.97, 85.78, and 71.92 cases per million persons per day, respectively. Based on the values of *ES*, it is suggested that the future trends regarding the pandemic in Brazil, Saudi Arabia, South Africa, and the USA are not optimistic and are full of challenges.

## 4. Discussion

The DEA in this paper shows that Korea, Australia, and Japan had better mitigation efficiency by 27 June 2020, while the USA, Brazil, and Russia performed less efficiently and were ranked at the bottom. Ahn [26] suggested that the successful experience in Korea to counter COVID-19 spread may be attributed to the mass testing and effective contact tracking system. Individuals testing positive for the infection after viral tests were hospitalized at special facilities. The people who had been in contact with the infected were to remain self-quarantined for 14 days. The availability of personal protective equipment was ensured to have a sufficient supply to avoid further infection at the onset of COVID-19 in Korea. In contrast, the testing capacity has not been sufficient to support the policies of a gradual reopening of the economy planned in many US states [27].

### 4.1. The Trend Patterns in Efficiency Ranks

The trend patterns in efficiency ranks also revealed information about future trends regarding epidemic mitigation. Type (1) and Type (2) countries may have more optimistic chances regarding recovery from the spread of COVID-19, as the efficiency ranks of Type (1) and Type (2) countries were high in Stage 6.

The Type (1) countries included the following seven countries: Korea, China, Italy, Spain, the UK, Germany, and France.

In addition to Korea, the other countries implemented effective responsive strategies, including extensive viral tests, lockdowns, social distancing, temporary cessation of sports events, school closures, and wearing of masks. In China, testing policies were promoted by expanding the testing of individuals from persons with symptoms to the open public on 12 February 2020, and all levels of school were closed on 26 January 2020 [28,29]. China has successfully slowed the transmission of COVID-19 through a combination of lockdowns, viral tests, contacting tracing, and other minor strategies, including street sanitization, school closures, and wearing of masks. Strict lockdowns and strict checks to avoid close contact between people were implemented in China after the outbreak. In less than three months, China gradually eased the strict policy of the lockdown and started to motivate the opening of economic activities. The strict lockdowns, wearing of masks, and social distancing implemented in China may be the major contributors to the effective prevention of transmission in a short time.

In contrast, the response of European countries such as Italy was not as prompt and urgent as that in Korea or China, and their efficiency ranks after Stage 4 were worse. For example, schools in Italy closed on 2 March 2020, and people were asked to stay at home, with exceptions for daily exercise and grocery shopping, on 23 February 2020. However, the testing policy adopted in Italy focused on testing anyone with COVID-19 symptoms after 26 February [28,29]. However, the efficiency ranks for the UK in the later stages (Stages 4–6) were much worse than those of other European countries. In March 2020, the UK attempted to reduce the impact of COVID-19 by means of herd immunity, but later, it denied the claims of herd immunity and argued that herd immunity is a natural by-product of an epidemic [30]. Given this situation, the strategy to fight against the epidemic was delayed, and thus, the effect was reduced.

Type (2) countries consisted of only Japan and Australia, with overall efficiency ranks of first and third place, respectively. In the middle stages, the efficiency ranks initially improved and then grew worse. A possible cause for these changes in efficiency ranks may be the low levels of viral testing in the earlier stages.

Extensive viral tests were performed in Australia and amounted to nearly 1000 tests per 100,000 people in the population by 31 March 2020 [31]. This number continued to increase and reached 2081 tests per 100,000 people on 28 April 2020 and 3119 tests per 100,000 people on 9 May 2020 (the final observation point in Stage 6 for Australia). The high testing rate in Australia may have been a major factor in mitigating the increase in new cases and leading it to have the best overall efficiency among these 23 countries.

In contrast, the trend in efficiency ranks for Type (3) countries showed a continual deterioration in mitigation efficiency. Compared to other countries, the coronavirus testing rate per capita in India was very low, reaching a total of 144,910 tests in a population with more than 1.3 billion people by 9 April 2020 [32]. On 14 May 2020 (the final observation point in Stage 6 for India), the viral testing rate was only 1.41 tests per 1000 people [29]. The low testing rate may be a key factor in explaining the good performance based on the high-efficiency ranking from Stage 1 to Stage 4. Without testing, no data are generated; thus, higher efficiency scores are obtained. As of 27 June 2020, the total number of confirmed cases in India reached 508,953, which was about 6.5 times the total number of confirmed cases of 78,003 during the entire period as of 14 May 2020.

At the onset of the outbreak, Russia announced a temporary ban on Chinese citizens from entering Russia on 20 February 2020 [25]. This strategy may have been effective in preventing infection through imported cases from China in Stage 1 and Stage 2. Extensive testing had been conducted in Russia, including 0.32 tests per 1000 people on 5 March 2020, 1.12 tests per 1000 people on 22 March 2020, 4.38 tests per 1000 people on 4 April 2020, 11.06 tests per 1000 people on 16 April 2020, 27.04 tests per 1000 people on 2 May 2020, and 45.61 tests per 1000 people on 16 May 2020 (the last day of Stage 6). However, Russia’s health department admitted that the test kits were often wrong and provided false-negative results. Therefore, the tested people with the virus were allowed to go home and thus infected other people. Thus, the real number of infected individuals was more than triple the official figure [33]. The ineffective tests may explain the continual deterioration in efficiency scores for Russia.

Type (4) countries contained the following nine countries: the USA, Iran, Turkey, Canada, Indonesia, Pakistan, South Africa, Argentina, and Brazil. If the current trends for these countries continue into the future, the outcomes do not look optimistic regarding the epidemic, and these countries need to devote more effort to improving mitigation in newly confirmed cases as their efficiency ranks were poor in the final stages. Some Type (4) countries lacked testing capacity in the earlier stages of the pandemic, and thus, the amount of testing that was performed was much lower than needed. Due to having less viral testing than the actual need, underestimation of newly confirmed cases may have taken place and led to the illusion of efficiency improvement, but eventually, efficiency ranks dropped in the final stages.

In the USA, the total number of tests performed relative to the size of the population before 7 March 2020 was very low, at less than 0.01 tests per 1000 people, and the situation gradually improved in March 2020 (in Stage 3). The testing rate increased to 0.23 tests per 1000 people by the end of March 2020 (in Stage 4) and then quickly increased to 10.43 tests per 1000 people on 16 April 2020 (in Stage 5). On the day of the final observation point in Stage 6 (7 May 2020), the testing rate rose to 24.63 tests per 1000 people, which seems to be a good figure compared to that of other countries. However, several experts have criticized the fact that the testing levels were not sufficient to meet the need for a gradual reopening by 1 May 2020 [27]. In addition, existing flaws in other response strategies also blocked improvements in the efficiency rank for the USA. For example, the US Centers for Disease Control and Prevention (CDC) emphasized the importance of mask-wearing, but Donald Trump continued to reject being photographed in public wearing a mask [34]. Some experts have suggested that the guidelines for mask-wearing have been confusing. Thus, many protesters across the country are described as people who refuse to wear a mask [35].

In fact, the USA has not been positively and seriously prepared for epidemic mitigation since the first confirmed case occurred on 23 January 2020. On 23 April 2020, Trump suggested injecting a powerful disinfectant into coronavirus patients as a possible cure for COVID-19. This news resulted in criticism from many scholars and reporters and disbelief and derision worldwide [36].

The trends in efficiency ranks for Type (5) countries, including Mexico, Nigeria, and Saudi Arabia, fluctuated more than those of the other country types. The testing rate in Mexico ranged from 0.01 to 3.1 tests per 1000 people during the whole period, which was much lower than that in other countries. Thus, the mitigation efficiency of Mexico ranked 17th among the 23 countries in Stage 5 and Stage 6. On 13 June 2020 (the final day of Stage 6 for Nigeria), the testing rate was 0.44 tests per 1000 people. Nigeria had a lower testing rate than Mexico, but the efficiency ranks for Nigeria were not bad. Thus, we reasonably suspect that the high-efficiency ranks of Nigeria may have been caused by an underestimation due to low viral testing rates.

### 4.2. The Correlation of Efficiency Ranks among Various Stages

Table 3 indicates that the correlation coefficient between two adjacent stages was higher than that between two non-adjacent stages. The correlation coefficients between Stage 1 and each stage after Stage 3 were low and negative. The negative or near-zero correlation coefficients between Stage 1 and Stages 4–6 imply that the efficiency ranking of the sampled countries at Stages 4–6 had been reorganized and completely differed from that at Stage 1. This implies that at Stage 1, some countries started to implement effective response strategies such as extensive viral testing, lockdowns, wearing of masks, etc., to prevent the spread of COVID-19 and thus created improved effects at Stages 4–6. In contrast, some countries purposely neglected the serious and emergent impacts arising from COVID-19 spread and failed to take any measures in response to the emergence of the epidemic. On the other hand, the high correlation coefficients between Stage 4 and Stage 5, Stage 4 and Stage 6, and Stage 5 and Stage 6 imply that the relative efficiency ranks among these countries became stable because their response strategies had stabilized.

The efficiency ranks in some countries showed a high degree of fluctuation across stages, especially the Type (5) countries. The high fluctuation in efficiency ranks implied that good efficiency rankings at a particular stage were only temporary and may have deteriorated in the next stage. The mitigation efficiency rankings for Type (3) countries continually worsened from Stage 1 to Stage 6. Thus, the Type (3) countries could not recover from the attack of COVID-19 in a short time and would have to adopt stricter response policies to mitigate the spread of COVID-19. Type (4) countries showed a U-shaped pattern, demonstrating temporarily improved ranks in the middle stages, but eventually, the ranking regressed in the final stages.

Both the inverted U-shaped (Type 1) and inverted N-shaped (Type 2) patterns in the trends in efficiency ranks seemed to be a good sign of improvement, as the efficiency ranks increased in the last stages. The probability of recovering from the attack of COVID-19 for Type (1) and (2) patterns is higher than that for other patterns. Nevertheless, the overall efficiency was calculated based on the whole period covering 105 days since the first confirmed case. The efficiency obtained was only temporary and could change for the better or worse if the assessment stage was extended to cover more days.

### 4.3. The Epidemic Stability

At the beginning of June 2020, the infectious disease COVID-19 remained a high risk in the world, but many countries have since attempted to lift the state of lockdown, restart the economy, and take action, as their governments have considered that the number of confirmed cases was greatly reduced and that newly diagnosed cases may be considered sporadic cases. For example, Trump attempted to end the lockdown and the stay-at-home order and to reopen schools at the beginning of June 2020 [37].

There was a high correlation between the efficiency scores in two adjacent stages, but it was still difficult to predict the epidemic stability of the next stage based on that of the previous stage. Thus, the data of the newly confirmed cases for the current dates are only for reference to determine the timing of restarting the economy. This paper suggests that an epidemic stability indicator in combination with a trend pattern of efficiency ranks such as Type (1) or (2) may be employed to judge the appropriateness of any measures to ease the response strategies such as travel restrictions, stay-at-home orders, and mask-wearing.

Low values of epidemic stability imply that the trend regarding the epidemic has attained a stable state and approached zero confirmed cases. Thus, China, Japan, Korea, and Australia seem to have recovered from the attack of COVID-19, while Brazil, Saudi Arabia, South Africa, and the USA remain engaged in the battle against COVID-19 and are required to devote more effort to create new opportunities. On 27 June 2020, China, Japan, Korea, and Australia had 24, 100, 51, and 37 daily new confirmed cases [1], respectively, being much lower than the peak of daily new confirmed cases for each country. In contrast, at the end of June 2020, Brazil and the USA continually set new records for daily new confirmed cases. The number of newly confirmed cases on 27 June 2020 was 39,483, 3938, 6215, and 40,526 cases for Brazil, Saudi Arabia, South Africa, and the USA, respectively [1].

On 30 June 2020, the European Council announced the easing of travel restrictions from 1 July 2020 for residents of recommended countries, including Australia, Japan, Korea, China, and Canada [38]. As indicated in Table 4, China, Japan, Korea, and Australia ranked first to fourth in epidemic stability. Canada was slightly behind in 13th place. To examine the appropriateness of lifting the travel restrictions at the external borders for residents of these countries, we used the data for 27 June 2020 as an example. On that day, the number of newly confirmed cases in China, Japan, Korea, Australia, and Canada was 24, 100, 51, 37, and 380, respectively, equivalent to a stability of 0.0168, 0.791, 0.995, 1.451, and 10.068 cases per million per day. The *ES* on 27 June 2020 in China, Japan, Korea, and Australia was much lower than the value of Germany’s *ES* (Table 4). This implies that the spread of COVID-19 had been controlled in these countries and was more stable than in Germany. The *ES* value on 27 June 2020 for Canada was nearly the same as that for France, as indicated in Table 4. However, Canada showed a U-shaped pattern for the trend in efficiency ranks, and it is suggested that the EU wait and observe the efficiency trend and the newly confirmed cases for Canada. Thus, the results suggest that the lifting of travel restrictions for these countries, with the exception of Canada, is quite reasonable based on the indicator of epidemic stability and the trends in efficiency ranking presented in this paper.

### 4.4. The Effect of Vaccination on Changes in Efficiency Ranks

Since the outbreak of the epidemic, many countries have devoted efforts to develop vaccines for COVID-19 to mitigate the transmission of the virus. As of April 2021, several vaccines were authorized by many countries for public use, including Pfizer-BioNTech, Moderna, BBIBP-CorV, CoronaVac, Covaxin, Sputnik V, AstraZeneca, and Johnson & Johnson [39]. By the end of May 2021, 1.17 billion doses of COVID-19 vaccines had been administered in the world [40]. The starting data of vaccination for these 23 countries and the cumulative vaccination rate by 25 May 2021 are listed in Table 5, according to which China and Russia started vaccinations earlier than other countries did, starting from 15 December 2020. Nigeria was the last country to start the vaccination program on 24 March 2021 among these 23 countries. 

Typically, individuals need two weeks after a one-dose vaccine or after vaccination of the second dose of a two-dose vaccine to have full protection against the COVID-19 virus [41]. Thus, the analysis of the effect of vaccination on efficiency change started from the beginning of 2021. The ranking of mitigation efficiency in the period of 4–17 January 2021 across countries was compared with that in the period of 10–23 May 2021. 

As of 25 May 2021, the UK and the USA had the highest cumulative vaccination rate, reaching 91.32 doses and 86.48 doses per 100 people indicated in Table 5, much more than that of other countries. In contrast, Nigeria and South Africa had only 0.94 doses and 1.18 doses per 100 people, ranking at the bottom. Table 5 also lists the mitigation efficiency rank for these countries in the period of 4–17 January 2021 (in terms of Rank1) and in the period of 10–23 May 20 (in terms of Rank2). Among these 23 countries, the efficiency rankings of the UK and the USA changed the most. After the start of the vaccination program, the efficiency of the UK and of the USA was greatly improved, from 23rd and 21st place in the period of 4–17 January 2021 to 9th and 13th place in the period of 10–23 May 2021, respectively.

The improvement in the ranking of mitigation efficiency in the United Kingdom and the United States can be explained by the substantial reduction in newly confirmed cases in these two countries. The reduction rate of newly confirmed cases in the UK was 97%, from 417,620 cases in the week of 4–10 January 2021 to 12,466 cases in the week of 17–23 May 2021. The newly confirmed case number in the USA decreased from 1,786,773 cases in the week of 4–10 January 2021 to 188,410 cases in the week of 17–23 May 2021, with a reduction rate of 89.45%. This implies that the vaccination programs implemented in these two countries have made a great contribution to protecting the public from infection. 

In contrast, the country with the most regressive efficiency ranking was India, which dropped from 7th place in the period of 4–17 January 2021 to 21st place in the period of 10–23 May 2021. The major reason for the efficiency drop in India may be the rapid increase in newly confirmed cases from 126,319 daily cases in the week of 4–10 January 2021 to 1,846,055 cases in the week of 17–23 May 2021. A possible cause for the surge in COVID-19 includes the easing of social distancing and mask-wearing as well as more human contacts due to mass political rallies for recent elections and religious events [42]. Compared to other countries, the vaccination rates in India were not bad, reaching 14.38 doses per 100 people, although this was much lower than the required herd immunity threshold of 65–70% vaccine coverage rates [43], equivalent to about 130–140 doses per 100 people. The case of the pandemic in India shows that the surge of the epidemic in a country is possible even if vaccination programs have been started.

Compared with the UK and the USA, the cumulative vaccination rate of European countries and Canada as of 25 May 2021, shown in Table 5, is 49.85 to 58.13 doses per 100 people, which is about 33–44% lower than that of the UK and the USA. However, the improvement in the mitigation efficiencies in these European countries and Canada is not so obvious as in the UK and the USA. This paper suggests that only when a country’s vaccination rate reaches a certain level can the spread of the epidemic be slowed down.

## 5. Conclusions

At the onset of the COVID-19 infection in different populations, mass testing programs and effective tracing systems on infected people were implemented in some countries, such as China and Korea. Based on the trends in efficiency ranks and the epidemic stability indicators, China, Korea, Japan, and Australia have performed better than other countries have. Thus, this paper suggests that mass testing together with other strategies such as contact tracing, lockdowns, mask-wearing, and social distancing are significantly effective in mitigating the transmission of COVID-19. Testing suspected persons identified through contact tracing and reducing interpersonal contacts through complete or partial lockdown also play important roles in reducing the number of confirmed cases. Castillo et al. [44] examined the effect of the stay-at-home policy on COVID-19 infection rates and found that the infection rate decreased from 0.113/day pre-policy to 0.047/day post-policy. Ferguson et al. [45] found that a lockdown may result in an average reduction in COVID-19 transmission by 50%, school closure by 20%, and other measures by approximately 10% (cited from Willis et al. [46]). Some other studies have also presented the same conclusions that non-pharmaceutical interventions may effectively prevent the spread of infection [47,48].

Pearson’s correlation tests were also performed in this paper to examine the impact of efficiency at earlier stages on that in subsequent stages and showed that the efficiency ranks for each country dramatically changed across stages. Due to insufficient testing facilities, the number of confirmed cases may be underestimated at the initial stages. Thus, the mitigation efficiency scores in the earlier stage might be less accurate. The main contribution of this paper is first that it demonstrates the relative mitigation efficiencies of various countries in various stages of the pandemic. Secondly, this paper integrates epidemic stability indicators with the obtained efficiency trends to judge the appropriateness of reopening the economy. While having not reached an appropriate level of epidemic stability, economic reopening may damage the anti-epidemic achievements from the earlier stages and lead to a second wave of the epidemic with exponential growth in the number of newly confirmed cases. In the future, a model needs to be developed to ensure the reliability of data on the number of confirmed cases reported by each country. In addition, the role of vaccines in affecting the spread of diseases may be worthy of attention.

## Figures and Tables

**Table 1:** The starting and ending dates of each stage for each country.

Country	Stage 1	Stage 2	Stage 3	Stage 4	Stage 5	Stage 6
China	2019/12/31–2020/1/30	2020/1/31–02/14	02/15–02/29	02/30–03/15	03/16–03/30	03/31–04/14
Japan	01/15–02/14	02/15–02/29	03/01–03/15	03/16–03/30	03/31–04/14	04/15–04/29
Korea	01/20–02/19	02/20–03/05	03/06–03/20	03/21–04/04	04/05–04/19	04/20–05/04
USA	01/23–02/22	02/23–03/08	03/09–03/23	03/24–04/07	04/08–04/22	04/23–05/07
Australia, France	01/25–02/24	02/25–03/10	03/11–03/25	03/26–04/09	04/10–04/24	04/25–05/09
Canada	01/27–02/26	02/27–03/12	03/13–03/27	03/28–04/11	04/12–04/26	04/27–05/11
Germany	01/28–02/27	02/28–03/13	03/14–03/28	03/29–04/12	04/13–04/27	04/28–05/12
India	01/30–02/29	03/01–03/15	03/16–03/30	03/31–04/14	04/15–04/29	04/30–05/14
Italy	01/31–03/01	03/02–03/16	03/17–03/31	04/01–04/15	04/16–04/30	05/01–05/15
Russia, Spain, UK	02/01–03/02	03/03–03/17	03/18–04/01	04/02–04/16	04/17–05/01	05/02–05/16
Iran	02/20–03/21	03/22–04/05	04/06–04/20	04/21–05/05	05/06–05/20	05/21–06/04
Brazil, Pakistan	02/27–03/28	03/29–04/12	04/13–04/27	04/28–05/12	05/13–05/27	05/28–06/11
Nigeria	02/28–03/29	03/30–04/13	04/14–04/28	04/29–05/13	05/14–05/28	05/29–06/12
Mexico	02/29–03/30	03/31–04/14	04/15–04/29	04/30–05/14	05/15–05/29	05/30–06/13
Indonesia	03/02–04/01	04/02/04/16	04/17–05/01	05/02–05/16	05/17–05/31	06/01–06/15
Saudi Arabia	03/03–04/02	04/03–04/17	04/18–05/02	05/03–05/17	05/18–06/01	06/02–06/16
Argentina	03/04–04/03	04/04–04/18	04/19–05/03	05/04–05/18	05/19–06/02	06/03–06/17
South Africa	03/06–04/05	04/06–04/20	04/21–05/05	05/06–05/20	05/21–06/04	06/05–06/19
Turkey	03/12–04/11	04/12–04/26	04/27–05/11	05/12–05/26	05/27–06/10	06/11–06/25

**Table 2:** Descriptive statistics of study variables.

Statistics	Total Confirmed Cases *n*	Population Density *d* (Person Per km^2^)	Urbanization Degree *u* (%)
Max.	1,193,452	527.30	92.00
Min.	6914	3.32	34.00
Average	190,093	151.40	71.48
Standard deviation	260,495	146.28	15.45

**Table 3:** Correlations of mitigation efficiency between different stages.

	Stage 1	Stage 2	Stage 3	Stage 4	Stage 5	Stage 6
Stage 1	1					
Stage 2	0.6739 ***	1				
Stage 3	0.4048 **	0.5666 ***	1			
Stage 4	−0.1433	0.0210	0.4297 **	1		
Stage 5	−0.0982	0.1918	0.2002	0.7884 ***	1	
Stage 6	−0.0824	0.1401	0.1783	0.7602 ***	0.9828 ***	1

**: *p* ≤ 0.05; ***: *p* ≤ 0.01.

**Table 4:** The epidemic stability for each country by rank.

DMU	$S_{0}$	$S_{f}$	Δt	*ES*	Rank
China	5.81	5.92	74	0.01	1
Japan	12.04	14.47	59	0.46	2
Korea	21.07	24.68	54	0.68	3
Australia	27.11	29.78	30	0.89	4
Nigeria	7.06	11.3	15	2.83	5
Indonesia	13.99	18.74	12	3.95	6
Germany	203.51	230.64	46	5.90	7
Italy	368.99	396.88	43	6.49	8
India	5.65	36.88	44	7.10	9
Spain	492.32	530.22	42	9.02	10
France	209.24	239.23	30	10.00	11
Turkey	227.25	230.63	2	16.92	12
Canada	180.16	271.9	47	19.52	13
Pakistan	54.12	90.04	16	22.45	14
UK	348.69	455.71	42	25.48	15
Iran	191.32	259.22	21	32.33	16
Mexico	103.91	157.41	14	38.21	17
Argentina	72.54	116.07	10	43.53	18
Russia	186.41	430.09	42	58.02	19
USA	360.56	727.36	51	71.92	20
South Africa	141.45	210.07	8	85.78	21
Saudi Arabia	379.3	501.46	13	93.97	22
Brazil	347.9	577.77	16	143.67	23

$S_{0}$: epidemic stability on the designated date (27 June 2020); $S_{f}$: the last day of Stage 6; Δt: the period between the designated date and the last day of Stage 6; *ES*: epidemic stability.

**Table 5:** The vaccination starting date, cumulative vaccination rates by 25 May 2021, and efficiency ranking in the weeks of 4–10 January (Rank1) and 17–23 May 2021 (Rank2).

Country	Vaccination Starting Date	Vaccination Rates ^#^ by 25 May 2021	Rank1	Rank2
UK	2021/1/3	91.32	23	9
USA	2020/12/20	86.48	21	13
Canada	2020/12/16	58.13	13	17
Germany	2020/12/27	56.52	14	16
Spain	2021/1/4	54.07	22	14
Italy	2020/12/27	53.54	17	15
France	2021/1/5	49.85	19	20
China	2020/12/15	39.37	1	1
Saudi Arabia	2021/1/6	38.49	3	8
Turkey	2021/1/14	33.81	15	10
Brazil	2021/1/19	30.38	18	22
Argentina	2020/12/31	25.07	16	23
Mexico	2020/12/27	21.49	11	7
Russia	2020/12/15	18.92	12	12
Australia	2021/2/23	14.88	2	3
India	2021/1/16	14.38	7	21
Korea	2021/2/26	11.79	6	4
Indonesia	2021/1/22	9.43	8	6
Japan	2021/2/22	8.38	9	11
Iran	2021/3/19	3.74	10	18
Pakistan	2021/3/14	2.43	5	5
South Africa	2021/2/19	1.18	20	19
Nigeria	2021/3/24	0.94	4	2

^#^ unit: doses per 100 people.

## Data Availability

The data for COVID-19 confirmed cases and vaccination rates can be found in WHO (https://www.who.int/emergencies/diseases/novel-coronavirus-2019/situation-reports, accessed on 18 June 2021) and Our World in Data (https://ourworldindata.org/covid-vaccinations, accessed on 18 June 2021), respectively.
